# Updating the Five Provisions: Aligning Welfare-Focused Care with the Five Domains Model

**DOI:** 10.3390/ani16121927

**Published:** 2026-06-22

**Authors:** Katherine E. Littlewood, Ngaio J. Beausoleil, David J. Mellor

**Affiliations:** 1 AkoVet Limited, Palmerston North 4410, New Zealand; 2 Animal Welfare Science and Bioethics Centre, School of Veterinary Science, Massey University, Palmerston North 4442, New Zealand; n.j.beausoleil@massey.ac.nz (N.J.B.); d.j.mellor@massey.ac.nz (D.J.M.)

**Keywords:** Five Domains Model, Five Provisions, welfare-focused care, contextualised care, good life, animal welfare, animal care, agency, welfare assessment, needs

## Abstract

The Five Domains Model has become one of the most widely adopted frameworks in animal welfare science and practice. The Model is now applied in a range of ways; among the most prominent are (1) as a framework for systematic and structured welfare assessment and (2) as an organising structure for planning and communicating appropriate (i.e., welfare-focused) care provisions, education, and standards. This paper focuses on these two applications and proposes a corresponding update to the affiliated Five Provisions and Welfare Aims. Specifically, we revise: (1) Provision 4 from “Appropriate Behaviour” to “Appropriate Choices” to reflect the 2020 update of the Model incorporating human–animal interactions and the 2023 operationalisation of agency in Domain 4; (2) Provision 2 from “Good Environment” to “Good Living Space” to resolve ambiguity with Domain 4’s “Interactions with the Environment”; and (3) Provision 5 from “Positive Mental Experiences” to “Integrated Care,” which captures consistent delivery of the first four provisions over time and across all those who interact with the animal. This update also pairs Provision 5 with a welfare aim that specifies the integrated mental state the animal should experience as a result. This change makes the distinction between care (provisions) and welfare (aims) consistent throughout the framework. It also makes explicit the integrative role of Provision 5, which parallels Domain 5’s role in the Model. We then describe the reasoning process that distinguishes welfare assessment from welfare-focused care provision. Welfare assessment uses the domain structure as a reasoning pathway, with the assessor using indicators and their impacts in Domains 1 to 4 to infer named mental (affective) experiences in Domain 5. Planning and communicating appropriate (i.e., welfare-focused) care uses the same structure to organise information about what is provided to animals, without executing the inferential step to Domain 5. Drawing on examples from organisations that use the Model for different purposes, we show that both applications are legitimate but produce different outputs. The Five Provisions framework, with its dual structure of provisions paired with welfare aims, serves the care planning and communication function more effectively than does the Model’s domain structure alone. Recognising these different uses also helps to locate where recent critiques of the Model apply and where they do not. Finally, we propose that the provisions and welfare aims framework can supplement “needs” language in legislation and policy to better reflect the distinction between animal care and animal welfare.

## Simple Summary

The Five Domains Model is a widely used framework for understanding and improving the lives of animals under human care. It is used both to assess how animals are experiencing their lives and to organise guidance on what people should provide. The planning and communication framework derived from the Model, the Five Provisions, has not been updated since 2016. Three of the five provisions’ names are now out of step with developments in the Model or with the framework’s own logic. This paper proposes three updates. First, Provision 4 is renamed from “Appropriate Behaviour” to “Appropriate Choices” to reflect the understanding that animals benefit from making meaningful choices and behaving accordingly. Second, Provision 2 is renamed from “Good Environment” to “Good Living Space” to resolve an ambiguity introduced by the 2020 revision of the Model. And third, Provision 5 is renamed from “Positive Mental Experiences” to “Integrated Care.” This reflects its structural role as the provision that brings the first four provisions together over time and across the range of people interacting with an animal. The mental state outcome Provision 5 supports, as specified in the paired welfare aim, is the minimisation of negative states and the promotion of positive ones. The paper also describes the difference between using the Model to assess an animal’s experiences and using it to describe what an animal is provided under appropriate (i.e., welfare-focused) human care. Both applications are valuable, but they are different. Being clear about which use is employed helps organisations to communicate accurately and ensures that providing good care is understood as a means to an end, with the animal’s actual experiences as the measure of success.

## 1. Introduction

The Five Domains Model has become one of the most widely adopted frameworks in animal welfare science and practice. First formulated in 1994 and progressively updated to reflect advances in understanding of animal sentience and affective experience, the Model is now used across sectors including farmed animal production, zoo and aquarium management, wildlife management, companion animal care, veterinary practice, and animal research [1,2]. At least 56 non-governmental organisations, industry bodies, and companies worldwide have adopted it as a framework for their approach to animal welfare, including the World Association of Zoos and Aquariums, the International Whaling Commission, and the Zoo and Aquarium Association Australasia [3]. Its influence extends to regulatory contexts, where it has been used by government departments to inform codes of welfare, evaluate housing systems, and compare the welfare impacts of management practices [4,5].

This widespread adoption means the Model is now used in a range of ways by different actors. Two prominent applications are the focus of this paper. In the first of these applications, the Model is employed to systematically assess animal welfare. Assessors gather evidence in the form of validated indicators across Domains 1 to 4, use that evidence to infer named mental (affective) experiences, and arrive at conclusions about the animal’s welfare status in Domain 5. In the second, more recent application, the Model’s domain structure is employed to organise and communicate welfare-focused care for animals, or to develop educational materials or standards focused on appropriate care provision. These latter applications do not draw inferences about what that care means for the animal’s mental experiences in Domain 5. These are all legitimate applications, but they engage with the Model in different ways and produce different outputs. Many organisations also reference the Model or declare alignment with it in policy documents and public communications without operationalising it in either case. For example, an organisation may state in a welfare policy that it is guided by the Five Domains Model without applying it either to assess welfare or to structure its care standards. Other uses of the Model exist; however, a fuller account of the range of applications is beyond the scope of this paper.

A framework derived from the Five Domains Model already exists for the second of these applications, i.e., for planning and communicating welfare-focused care. The Five Provisions and Welfare Aims [6,7,8] translate the Model’s structure into goal-directed, practical guidance for those responsible for animal care, while preserving the distinction between what is provided to the animal (care) and what the animal experiences (welfare). Animal care refers to the resources, conditions, and management practices provided to animals. Animal welfare, understood in affective state terms, refers to the animal’s mental experiences: the negative and positive affective states that arise from physical/functional states or available conditions in Domains 1 to 4 and that are inferred in Domain 5 [6,8,9]. Care is the input. Welfare is the outcome for the animal. Good care is necessary for good welfare, but it is neither sufficient nor synonymous with it, and the two are not interchangeable. Each of the Five Provisions names a category of care input, and each is paired with a Welfare Aim specifying the affective outcome the input is intended to support. This dual structure makes the Five Provisions framework better suited to planning and communicating welfare-focused care than the Model’s domain structure. However, the Five Provisions framework has not been updated since 2016, and developments in the Model and in welfare science since then call for the corresponding updates we set out here.

This paper has two aims. First, we propose an update to the Five Provisions and Welfare Aims [6,7,8], the planning and communication framework derived from the Five Domains Model. We revise Provision 4 from “Appropriate Behaviour” to “Appropriate Choices” to reflect the 2020 revision of the Model [2] and the operationalisation of agency within Domain 4 through the qualities of choice, control, and challenge [10]. In addition, we revise Provision 2 from “Good Environment” to “Good Living Space”. And finally, we revise Provision 5 from “Positive Mental Experiences” to “Integrated Care” to capture a dimension of care not otherwise reflected (the integrated delivery of the first four provisions), and pair it with a welfare aim that names the affective outcome. This makes the distinction between care and welfare consistent across the framework, and makes Provision 5’s integrative role explicit. Second, we distinguish two prominent uses of the Five Domains Model: (1) as a framework for systematic welfare assessment, and (2) as an organising structure for planning and communicating the care provisions required to support good welfare (i.e., so-called “welfare-focused care”). We describe the reasoning pathway that distinguishes welfare assessment from planning and communicating care. Finally, we explain how the Five Provisions framework, with its dual structure of provisions paired with welfare aims, serves the welfare-focused care planning and communication function more effectively than does the Model’s domain structure alone.

## 2. The Five Provisions and Welfare Aims: Origin and Purpose

The Five Provisions and Welfare Aims were introduced by Mellor in 2016 [6] as a successor to the Five Freedoms [11,12,13]. The Five Freedoms had been enormously influential in establishing the scope of animal welfare concern, but their framing, in terms of freedom from negative states, limited their conceptual reach. They were structurally oriented toward preventing all degrees of all negative experiences, with no framework for recognising or promoting positive ones [6,7,8].

The Five Provisions addressed this by aligning more practical management targets with the domain structure of the Five Domains Model [6,8]. The first four Provisions, designated Good Nutrition (Provision 1), Good Environment (Provision 2), Good Health (Provision 3), and Appropriate Behaviour (Provision 4), correspond to Domains 1 to 4 and describe what should be provided. Provision 5, Positive Mental Experiences, corresponds to Domain 5 and describes the overall aim: that the animal’s mental state should be characterised by a predominance of positive experiences [6].

This overall aim sits at the upper (aspirational) end of the quality of life continuum, which ranges from a life not worth living to a good life, with the categories defined by the balance of positive and negative experiences the animal has. The concepts of a life worth living and a good life were introduced by the Farm Animal Welfare Council [14] and incorporated into the Five Domains lineage through the updated Provisions [6,15]. Therefore, the Five Provisions exist to support a good life for the animal, understood as a life in which positive experiences predominate, rather than provision as an end in itself. On this understanding, the Five Provisions can be read as principles for providing a good life. Each names a category of care that, delivered together and sustained over time, creates the conditions in which positive experiences can predominate.

Each of the first four Provisions was paired with a Welfare Aim specifying the intended affective outcome for the animal so provisioned. The Welfare Aim for Provision 1, for example, is to “Minimise thirst and hunger and enable eating to be a pleasurable experience.” The Welfare Aim for Provision 3 is to “Minimise breathlessness, nausea, pain and other aversive experiences and promote the pleasures of robustness, vigour, strength and well-coordinated physical activity” [6]. This dual structure, provision plus welfare aim, was designed to make explicit that providing resources and conditions (care) is a means to an end, and that the end is the animal’s affective experience (welfare). Therefore, the Five Provisions framework is a planning and communication tool derived from the Five Domains Model. It is not a welfare assessment framework. It translates the Model’s structure into practical guidance for those responsible for animal care, while preserving the distinction between what is provided and what the animal experiences (i.e., welfare-focused care) [6].

The relationship between the Five Provisions and the Five Domains Model is closer than structural derivation alone would explain. Both frameworks are explicitly oriented toward the animal’s mental experiences as the primary concern. In the Model, this is expressed through the reasoning process used to infer named mental experiences in Domain 5 from physical/functional states or available conditions across Domains 1 to 4. In the Provisions, this is expressed through the welfare aims, which specify what the animal should experience as a result of the care provided (i.e., it provides a structure for welfare-focused care). Whether the framework of interest is the Model or the Provisions, the animal’s mental experiences serve as the reference point [2,6].

This affective state orientation, which places animals’ subjective experiences at the centre of welfare evaluation and care provision, is consistent with the broader shift in animal welfare science away from biological functioning as the primary indicator of welfare status and toward what animals actually experience [2,16]. It also reflects the way many people intuitively understand good welfare: not simply as the presence of good conditions, but as what the animal experiences in those conditions. The widespread adoption of the Model across sectors reflects this alignment between the framework’s orientation and the underlying concern that drives many organisations and individuals to engage with animal welfare.

This orientation is also what genuine engagement with both frameworks requires. The domain structure of the Model can be used as a set of category headers, and the provisions as a list of care inputs, without engaging with the underlying commitment to animal-centred welfare assessment or animal-centred care. When that happens, the frameworks are invoked rather than applied, and care provision may inadvertently become the endpoint rather than the means to a positive affective (welfare) outcome. The welfare aims are designed to make this distinction visible: they direct attention to what the animal should be experiencing, not only to what has been provided.

## 3. Updating the Provisions

### 3.1. Updating Provision 4 from “Appropriate Behaviour” to “Appropriate Choices”

Since the inception of the Five Provisions in 2016, two key developments have occurred that necessitate a revision to Provision 4: the 2020 update to the Five Domains Model and the 2023 operationalisation of agency in its fourth Domain [2,10]. The 2020 update incorporated human–animal interactions as a distinct element within Domain 4 [2]. This revision recognised that interactions with people are a significant and unavoidable influence on the welfare of most animals under human care. The revision also formalised the three categories of interaction assessed within Domain 4: interactions with the environment, interactions with other animals, and interactions with people [2]. However, the original “Appropriate Behaviour” designation for Provision 4 does not clearly signal these categories [6].

The concept of agency has been recognised as central to Domain 4 since the 2015 revision of the Model. Mellor and Beausoleil (2015) identified that an animal exercises agency when it engages in voluntary, self-generated, and goal-directed behaviours [17], drawing on Wemelsfelder (1997) and Špinka and Wemelsfelder (2011), and linked agency to the concept of positive affective engagement [18,19]. The 2020 revision maintained this framing [2]. Littlewood et al. (2023) further operationalised the concept for welfare assessment, identifying three agentic (agency-related) qualities through which agency can be assessed: choice, control, and challenge, and showing how these link to specific affective experiences [10].

This operationalisation sits within a broader body of work on the welfare significance of choice and control. Leotti et al. (2010) provided evidence that the perception of control over one’s environment is an affective and biological necessity across species, not merely a desirable condition, and that choice is the means through which control is exercised [20]. Špinka (2019) proposed four levels of agency, from passive/reactive to aspirational, and argued that competence-building agency, in which animals engage with the environment to gain skills and information, is a central adaptive characteristic with direct welfare implications [21]. Englund and Cronin (2023) reviewed the intersection of choice, control, and welfare, arguing that the benefits of environmental control are mediated through the availability of meaningful choices [22]. This convergence across independent research programmes supports the position that choice is not merely one component of agency but its primary vehicle and distinguishes agency from behaviour more generally: agency refers specifically to behaviour that is volitional, purposive, and intrinsically motivated.

The operationalisation of agency shifts attention from what the animal does (behavioural output) to whether the animal has genuine opportunities to act on its own motivations. A recent scoping review of experimental studies comparing choice to non-choice conditions found that most studies reported improved behavioural and physiological welfare indicators when animals were provided with the ability to select among available options, although the authors noted that the empirical evidence base remains small and taxonomically narrow [23]. When animals can exercise agency, the resulting engagement is associated with positive affective states, including “positive affective engagement”, a state reflecting the pleasurable occupation inherent in voluntary, goal-directed activity [2,10,17,24]. The original title of Provision 4, “Appropriate Behaviour”, does not capture this. An animal may exhibit behaviour without exercising agency, for example, when behaviour is reactive, compulsive, or constrained to a narrow range of options that do not reflect the animal’s motivational priorities.

#### 3.1.1. The Intent Behind “Appropriate Choices”

“Choices” captures the concept of agency: the animal as an active agent exercising voluntary, goal-directed behaviour among available options. The word positions the animal as a decision-maker rather than a behavioural performer, aligning with the conceptual shift from behaviour-as-output to agency-as-capacity [2,10,17,18,19,24].

“Appropriate” is not a generic modifier. It specifies two conditions under which choice is welfare-relevant. First, appropriate choices involve genuine control: the animal’s actions must lead to meaningful outcomes. Options that do not lead to different consequences are not genuine choices [10]. An animal whose attempts to modify their environment produce no effect is not exercising meaningful choice, regardless of the range of behaviour they display. Second, appropriate choices involve an appropriate level of challenge: the complexity of available choices should be calibrated to the animal’s cognitive and physical capacities [10]. Choices that are trivially simple do not engage the animal’s agency in a welfare-relevant way. Choices that are impossibly complex may generate frustration rather than positive engagement [10]. This calibration of challenge to capacity is consistent with the broader literature on the welfare value of environmental control. Perceived control, whether exercised or not, influences affective state [20,22]. The welfare value of choice is therefore not simply a function of the number of options available but of whether those options afford genuine control over outcomes that matter to the animal. This revision also maintains continuity with the original provisions framework. Mellor (2016) used “Appropriate Behaviour” for Provision 4 [6]. We retain the qualifier “Appropriate” and change the operative noun from “Behaviour” to “Choices.” This signals an update within the same framework, reflecting the application of evolving scientific knowledge, rather than a departure from it.

The three interaction categories of Domain 4 of the Model (environment, other animals, people) are not displaced by this revision [2]. Rather, they represent the contexts in which appropriate choices are exercised. An animal makes choices about how to interact with their environment, with other animals, and with people. The associated provision header focuses on the nature of what the animal does (i.e., exercises choice) rather than on what they interact with. This is reflected in the Provide column for Provision 4 (Table 1), which is structured around the three subcategories. “Appropriately varied and explorable spaces” addresses choices about how to interact with the environment. “Opportunities for compatible social contact” addresses choices about interactions with other animals, including conspecifics and compatible interspecific companions. “Predictable human interactions the animal can influence or avoid” addresses choices about interactions with people: it is shorthand for considerate human interactions in which the animal has some capacity to opt out, signal preferences, or modify the encounter. In each case, the “Provide” content names the opportunities the caregiver creates; whether the animal exercises agency through these affordances depends on the animal. The Provide column lists the principal contexts, but is not exhaustive. For example, the Provide column does not name food-choice or feed-foraging choices, even though these are potential expressions of agency.

“Choices” rather than “Agency” was selected for two reasons. First, within the Five Provisions and Aims framework, provisions describe what to provide, and the concept of affordances clarifies why these matter. An affordance is what an environment offers an animal, measured relative to that animal rather than in abstract physical properties [10,25]. A person responsible for an animal’s care can create conditions that afford genuine choices: varied environments, compatible social groupings, and predictable and positive interactions with people. Whether the animal exercises agency through those affordances depends on the animal’s perceptual world, their capacities, and their motivational state [10,25]. The provision creates affordances; the animal’s perception of and engagement with those affordances determines the welfare impact. “Appropriate Choices” sits on the care side of this distinction, which is where a provision header belongs. “Agency” sits on the welfare side, i.e., the aligned Aim. Second, “choices” is readily understood by a general audience, whereas “agency” is a technical term that requires explanation. As the Five Provisions are largely a planning and communication framework, the headers should be framed for a general audience.

The corresponding Welfare Aim for the updated Provision 4 is to: “Minimise fear, anxiety, and other unpleasant experiences and promote the pleasure of making meaningful choices and acting on them, including interest, engagement, and a sense of control.” Two features of this revision are worth noting. First, “threats” has been removed from the minimise clause. “Threats” describes a feature of the conditions provided rather than an affective outcome the animal experiences, and including it in a Welfare Aim was structurally inconsistent with the form of the other Welfare Aims. The revised minimise clause names the affective consequences (fear, anxiety, and other unpleasant mental experiences) that restrictions on agency, particularly the ability to avoid stimuli or conditions perceived as aversive, generate. Second, the promote clause now names the specific positive experiences associated with the operationalisation of agency through choice, control, and challenge: interest, engagement, and a sense of control.

#### 3.1.2. Corroboration from the Five Freedoms Lineage

The shift towards agency-informed framing is not confined to the Five Domains Model literature. John Webster, who originated the Five Freedoms [12], has recently revised the fifth freedom from “Freedom to exhibit natural behaviour” to “Freedom of choice” [13,26]. His reasoning is explicit: that the original formulation gave insufficient recognition to the quality of life of animals, whereas “choice” gives greater respect to the principle of autonomy. His revised formulation recognises that animals should be able to make a positive contribution to their own quality of life through their own actions [26].

This is an independent convergence on the same point. However, there is a limit to how far Webster’s revision goes. “Freedom of choice” remains structurally a freedom-based framing. It does not reach the operationalised account of agency in Littlewood et al. (2023), in which agency is assessed through the qualities of choice, control, and challenge [10]. “Appropriate Choices” goes further by specifying the conditions under which choice is welfare-relevant, involving genuine control and appropriate challenge. The animal is positioned as an active agent rather than a beneficiary of freedom.

### 3.2. Updating Provision 2 from “Good Environment” to “Good Living Space”

The 2020 update of the Five543 Domains Model renamed Domain 2 from “Environment” to “Physical Environment” to distinguish it from “interactions with the environment” assessed within Domain 4 [2]. The same ambiguity exists in the provisions framework. “Good Environment” could be interpreted as encompassing Domain 4 interactions, which belong under Provision 4. “Good Living Space” is more concrete and is readily understood by a general audience. Living space signals the physical and sensory conditions covered by Domain 2: the space and the conditions within it, including air quality, lighting, temperature, substrate, and resting areas, not only its spatial extent. The revised Welfare Aim, paired with Provision 2, addresses aspects the header does not signal directly, such as visual, olfactory, auditory, magnetic, vibrational, tactile, and other comforts: “Minimise discomfort and promote physical, thermal, and sensory comforts” (Table 1).

### 3.3. Updating Provision 5 from “Positive Mental Experiences” to “Integrated Care”

The original name for Provision 5 is inconsistent with the logic that differentiates care and welfare throughout the framework. Provisions 1 to 4 follow a consistent pattern: each names a category of care input (nutrition, living space, health, choices) and is paired with a welfare aim specifying the affective outcome those inputs are intended to support. This dual structure makes the care/welfare distinction explicit. The provision is what the caregiver provides; the welfare aim describes what the animal should experience as a result.

“Positive Mental Experiences” does not fit this pattern. Positive mental experiences are not a care input that can be provided. They are what the animal has, or does not have, depending on whether the care delivered across the first four provisions is of sufficient quality and appropriately integrated. Naming them as a provision inadvertently frames an affective outcome as a care provision, a conflation that the framework’s dual structure is designed to prevent.

The updated Provision 5 is “Integrated Care.” It is structurally different from the other four Provisions, each of which names a distinct category of care input. Provision 5 does not name a fifth category of care; it specifies that the first four are delivered consistently over time and across all those who interact with the animal. This parallels the role of Domain 5 in the Model. Domain 5 is not a fifth category of physical, functional, or agentic (agency-related) condition; it is the integrative domain, where the affective consequences of conditions across Domains 1 to 4 are drawn together, and overall welfare status is inferred [2]. Domain 5 has no indicators of its own. It is inferred from evidence gathered across Domains 1–4. Provision 5 works the same way: it does not add a fifth category of care but specifies how the first four are delivered. Naming Provision 5 “Integrated Care” makes this integrative role visible in the name itself, rather than presenting it as a fifth co-equal care category.

“Over time” encompasses two related but distinct requirements. The first is sustained provision within the animal’s life: nutrition, living space, health care, and choices must be consistently provided rather than supplied at a single point and then withdrawn. The second is responsiveness to life stage and to individual variation in capacities, past experiences, and interests. For example, the substance of each provision may change as the animal matures, ages, or undergoes physiological changes such as gestation, lactation, weaning, recovery from illness or injury, or senescence. What constitutes Good Nutrition for a juvenile is not what constitutes it for an aged individual. Appropriate Choices for a young animal may differ from Appropriate Choices for one approaching the end of its life or among animals with different capacities, past experiences, or interests. Past experiences in particular shape what an animal perceives as aversive and seeks to avoid. Therefore, integration over time means both temporal consistency and life-stage adaptation. Adapting the substance of each provision to the individual animal in this way is consistent with the move in veterinary practice toward contextualised care [27]. Sustained provision does not require that every provision be fully delivered at every moment. Some periods of partial provision are unavoidable, such as fasting and restricted movement during transport. Others serve the animal’s interest, such as the temporary restriction of agency entailed in cage rest during recovery from injury. The standard is the integrated pattern of care across the animal’s life, with the affective balance specified in Welfare Aim 5 as the measure of whether that integration is adequate (i.e., welfare-focused care). This connects Provision 5 to the broader concept of a life worth living [6,15]: a life of acceptable welfare is sustained across its full duration and adjusts to the animal’s changing capacities and requirements.

“Across all those who interact with the animal” acknowledges that most animals in human care are cared for by multiple people, almost always concurrently and often at different stages in their lives. Many jurisdictions impose a legal duty of care on those who are “responsible for” an animal. Provision 5 extends this responsibility, in substance if not in law, to consistency in how that care is delivered by everyone who interacts with the animal over the course of their life. Owners, family members, professional caregivers, veterinary staff, trainers, handlers, groomers, farm staff, transporters, rural contractors, and other service providers may all contribute to the care of the same animal. Concurrent caregivers may apply different standards if they are not coordinated. Sequential caregiving, as occurs during rehoming or transfers between facilities, creates points where standards may not carry over. Therefore, “Integrated care” implies consistency in how provisions are delivered across the people who interact with an animal: deliberate communication, shared understanding of what is required, shared training, and shared accountability. This is sometimes described as “a culture of care” [28,29]. A culture of care orients everyone who interacts with an animal toward the consistent application of care (the four provisions), regardless of which individual is delivering care at any given moment. Therefore, the integrative function of Provision 5 extends beyond the individual caregiver’s diligence to the relational and institutional context in which care is delivered.

**Figure 1:**
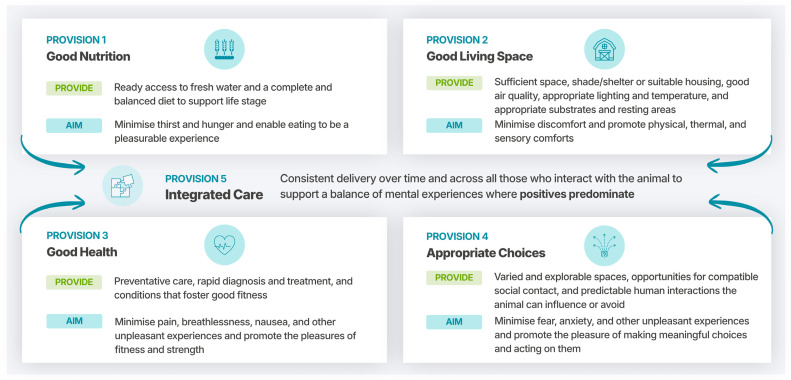
The updated Five Provisions and Welfare Aims. Each provision names a category of care input and is paired with a welfare aim specifying the affective outcome that input is intended to support for welfare-focused care. Provision 5 (Integrated Care) describes how the first four provisions should be delivered: consistently, over time, and across all those who interact with the animal. The welfare aim for Provision 5 specifies that the purpose of this integration is to support a balance in which positive experiences predominate. Arrows from Provisions 1–4 converge on Provision 5 to indicate that Integrated Care is not a separate input but the consistent, coordinated delivery of the first four provisions.

The Welfare Aim for Provision 5 represents the affective content that the original name was intended to convey, now repositioned where it belongs within the framework. The new Welfare Aim is: “Support a balance of mental experiences where positives pre-dominate.” This positions the animal’s mental experiences as the outcome of integrated care (i.e., welfare-focused care), not as care itself. This balance is the affective standard that defines a good life at the upper end of the quality of life continuum; Welfare Aim 5 names that standard, and the first four provisions are the means by which it is reached. The animal’s overall experience over time, situation and caregivers, not the completeness of the provision checklist, is the standard against which integration is judged. Table 1 presents the updated Five Provisions and Welfare Aims framework. Table 2 summarises the substantive revisions to the Five Provisions and Welfare Aims, listing the original and updated names and the rationale for each revision. Figure 1 presents the updated framework in full.

## 4. The Five Domains Model in Practice: Welfare Assessment (Reasoning) Versus Welfare-Focused Care (Planning and Communication)

The Five Domains Model is used in a wide range of contexts, from regulatory decision-making to certification standards to public-facing communication [3]. These applications differ in a fundamental way that relates directly to the distinction between care and welfare discussed above. Some use the Model to systematically assess welfare (outcomes), while others use the Model’s domain structure to organise, plan, and communicate care provisions (inputs) expected to result in good welfare (i.e., welfare-focused care). Both are legitimate and useful, but they are distinct applications with different purposes, processes and outputs.

This section proceeds in three steps. Section 4.1 and Section 4.2 set out the two main uses of the Model: (1) systematic welfare assessment, and (2) planning and communicating welfare-focused care. Section 4.3 uses that distinction to locate where recent critiques of the Model apply. Section 4.4 then draws out the implications, beginning with practice and turning to welfare science and to legislation and policy.

**Figure 2:**
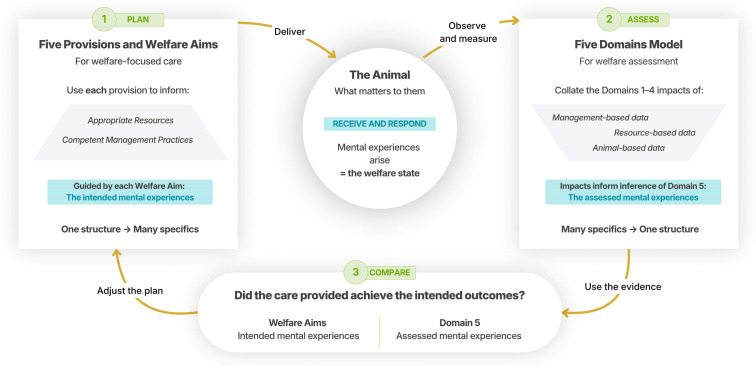
How the Five Provisions and the Five Domains Model work together, and how they differ. The two frameworks both focus on what matters to the animal (i.e., their mental experiences). They may be used together across a single cycle of care provision and adjustment, to: (1) plan and deliver appropriate welfare-focused care (i.e., using the Five Provisions and Welfare Aims); (2) collate data about animals and assess their welfare (i.e., using the Five Domains Model); and (3) compare outcomes against the welfare aims and adjust the plan (i.e., using both).

Figure 2 shows how the two frameworks relate across a single cycle of care and where they differ. The Provisions can be used to design and deliver welfare-focused care; the Model can be used to assess what the animal experienced; and the two are compared to judge whether the care achieved its intended outcomes for the animal.

These two applications do not exhaust the ways the Five Domains Model is used. The Model also appears in policy statements as a signal of commitment to welfare-centred care. The distinction this paper draws applies most directly to welfare assessment and care provision, where the difference between what is provided and what the animal experiences determines what can be accurately claimed.

Welfare-focused care is planned under each provision as a collection of appropriate resources and competent management practices, and is guided by the Welfare Aim (i.e., the intended mental experiences). The care is delivered; the animal receives it and responds (i.e., their mental experiences arise). The animal’s welfare is assessed using the Five Domains Model (i.e., the assessed mental experiences). Observations and measurements (animal-based, resource-based, and management-based data) are organised in Domains 1 to 4 of the Model to reflect physical/functional/agentic states. Domain 5 of the Model is then inferred from that evidence as the assessed mental experiences. Comparing the assessed mental experiences in Domain 5 (i.e., from the Five Domains Model) with the intended mental experiences in the Welfare Aims (i.e., from the Five Provisions) shows whether the care achieved what it was designed to do, and informs adjustments to the plan.

### 4.1. The Reasoning Process Underpinning Welfare Assessment Using the Model

When the Five Domains Model is used for systematic welfare assessment, the assessor engages in a structured reasoning process [3,30,31]. The process requires the assessor to collate species-specific data across Domains 1 to 4 using animal-based, resource-based, and management-based indicators measured using agreed scientific methods. For each indicator, the assessor first identifies the physical, functional, or agentic (agency-related) impact the indicator may reflect. For example, a low body condition score may indicate inadequate nutritional intake; pacing in a barren environment may indicate constrained agency. The assessor then completes what has been termed the ‘affective translation’ step: inferring, for each identified physical, functional or agentic impact, the specific mental experience it is likely to reflect. There is value in naming the specific quality of the affective experiences aligned with the observed impact, as far as is justified by available knowledge; for example, hunger, frustration, boredom, pain, comfort or positive affective engagement rather than “negative welfare” or “compromised welfare”, “stress” or “distress”. Potential benefits of this specific naming include emphasising the importance of the mental experience to the animal itself, engendering empathy, demonstrating the credibility of the inference based on an understanding of the underlying cause, and suggesting targeted strategies to mitigate unpleasant experiences or promote positive ones [32].

This two-step inference chain, from indicator to physical/functional/agentic impact, and then from impact to named affective experience, is the defining feature of welfare assessment using the Five Domains Model [31]. However, the weight of the three indicator types in this inferential process is not equivalent. Resource-based and management-based indicators function only as alerting indicators: they identify conditions under which particular affective states may occur, but they do not confirm that a specific experience is occurring for the focal animal(s). Animal-based indicators, when interpreted through the two-step inference chain, provide stronger evidence that a named experience is occurring for the focal animal(s). To illustrate, the absence of water (a resource-based indicator) alerts the assessor to the risk of current or future thirst, whereas water-seeking behaviour or a high score on a skin-turgor test (both animal-based indicators) indicates that thirst is actually occurring [30].

An assessment built primarily on resource- and management-based indicators can identify conditions or practices that are consistent or inconsistent with acceptable welfare; its conclusions concern the environment and management, not the animal’s current affective experience. This is the basis for the care/welfare boundary drawn throughout this paper: an assessment that does not include validated animal-based indicators and that does not complete the affective translation step describes conditions (care) rather than welfare, regardless of how systematically those conditions have been documented. Completing this chain for all relevant indicators across all four domains and then integrating the resulting affective inferences into a Domain 5 conclusion about the animal’s multifaceted mental experiences produces a detailed welfare assessment. It is this step, the affective translation and its integration in Domain 5, that distinguishes welfare assessment from a description of welfare-focused care.

This reasoning process has been applied rigorously in a number of contexts, particularly in the Australasian region where the methodology was developed. The New Zealand National Animal Welfare Advisory Committee used the Model to evaluate the relative risk of welfare impacts for different farrowing and mating systems for pigs. The output informed regulatory decisions under the Animal Welfare Act 1999 as part of its review of the Code of Welfare for Pigs [4]. Likewise, Sharp and Saunders (2011) in Australia, Beausoleil et al. (2016) in New Zealand and Baker et al. (2022) and de Ruyver et al. (2023) in the UK used the Model in this way to assess and compare the relative welfare impacts of different vertebrate pest control methods, generating profiles that allowed identification of less harmful approaches [5,33,34,35]. Colloff et al. (2024) used a similar process to evaluate options for blood sampling wild badgers [36], while Boys et al. (2022) and Serres et al. (2024) applied the Model to assess the welfare of stranded or free-living cetaceans [37,38,39]. Harvey et al. (2020) applied the Model to free-living horses, reaching conclusions about the likely affective state of individual animals to inform management decisions [30].

In each case, the application goes beyond merely describing the available conditions to which the animals are exposed. It assesses the likely affective consequences and infers them in Domain 5, ideally accompanied by an indication of the assessors’ confidence in the evidence on which the inferences are based [33]. The reasoning process is actively engaged and completed. However, the Model’s wider international adoption has mainly focused on planning or communicating care provisions rather than on this kind of systematic welfare assessment, highlighting the importance of a well-structured framework for that purpose.

### 4.2. Using the Model to Plan and Communicate Welfare-Focused Care Provisions

Other applications use the Five Domains Model’s domain structure to organise care standards or to communicate what is or should be provided to animals, without explicitly engaging with the reasoning process or completing the affective translation to Domain 5. In each, the domain structure is repurposed for a function it was not designed to perform: planning or communicating care (inputs) rather than assessing welfare (outcomes). The Five Provisions framework was developed for this function, and these examples show where and why it would be the more appropriate tool.

SPCA New Zealand’s Certified^®^ programme uses the Five Domains structure to organise its animal welfare standards for farms and companion animal care businesses. SPCA states that its standards are derived from the Five Domains of animal welfare and are based on both reducing negative experiences and providing positive experiences [40]. The standards specify what must be provided in terms of nutrition, physical environment, health, and behavioural interactions to achieve certification. The domain structure organises the care requirements; however, the affective translation is not completed, and no Domain 5 conclusion is derived from the evidence. This ensures comprehensive coverage of care provisions and goes beyond minimum legal requirements.

A common feature of this kind of application is that the four input-side domains (Nutrition, Physical Environment, Health, and Behavioural Interactions) are operationalised through requirements specified at the standard level, but Domain 5 is not. This is appropriate because Domain 5 has no independent indicators: it is the integrated affective outcome inferred from evidence across the other domains [17,31]. SPCA New Zealand addresses the affective dimension (Domain 5) by selecting specific requirements from Domains 1 to 4 that are expected to support positive mental experiences and minimise negative experiences. The affective intent is then stated in the preamble or rationale rather than embedded in the standards themselves (i.e., that the specific requirements support welfare-focused care).

The World Association of Zoos and Aquariums (WAZA) has adopted the Five Domains Model as the basis for its Animal Welfare Strategy and recommends that member institutions apply the Model to assess animal welfare [41]. In practice, many member institutions use the domain structure to organise their care standards and communicate their welfare commitments. Wellington Zoo, for example, describes its approach to animal care as using the Five Domains of animal welfare “to ensure their animals are happy and healthy” [42], with veterinary care, keeper care, and engagement opportunities organised under the Model’s domain headings. The Zoo conducts internal welfare assessments of individual animals using the Model, and its public-facing communication describes care provisions organised by domain.

The same care planning or communication application appears across other sectors. In textiles, it has been adopted in Textile Exchange’s Materials Matter Standard [43] and a FOUR PAWS’ 2025 review of certification standards [44]. It also appears in pedagogical applications, including SPCA New Zealand’s Kids Education site [45], NZ Pony Club [46], Thoroughbred Racing New Zealand [47], other SPCAs internationally, and veterinary curricula and continuing professional development courses. In each case, the domain structure is used to organise what should be provided rather than to assess welfare.

A risk inherent in the four-domain care-framing approach is that the connection to the animal’s mental experience, when not made explicit in the standards themselves, may fade from view over time. As personnel change and standards are revised, the connection between specified care inputs and intended mental experiences can become tacit, leaving the standards functioning as a checklist of inputs rather than a welfare-focused tool oriented to the animal’s experience.

The Provisions and Aims framework addresses this directly. Each Provision names a category of care input, paired with a Welfare Aim that names the affective outcome the input is intended to support. The affective dimension is not left as an implicit assumption or relegated to a preamble; it is part of the standard itself. Together, the provisions and welfare aims are goal-directed and focus the user’s attention on understanding how, and to what extent, the care provided achieves goals framed in terms of the animal’s own experiences (i.e., the extent to which the care is welfare-focused).

### 4.3. Locating Recent Critiques of the Five Domains Model

The distinction between welfare assessment (Section 4.1) and welfare-focused care (Section 4.2) also helps to understand where recent critiques of the Five Domains Model apply and where they do not. Hampton et al. (2023) raised concerns about aggregation, scoring, the subjective nature of grading welfare impacts, and difficulties of inferring animals’ affects from observable or measurable indicators [48,49]. These concerns are not uniform in their target, nor are many unique to the Five Domains Model. Some relate to specific applications of the Model, particularly protocols for scoring relative welfare impacts in wildlife management contexts and other settings where the Model is used to compare options or rank outcomes. Others, such as the difficulty of validating affective inferences and of aggregating various indicators to reflect the overall welfare state, apply more broadly to welfare assessment using multi-dimensional approaches and represent genuine challenges for the field [50]. These concerns can largely be addressed through careful reporting of process, evidence, and assessor confidence [34]. None of these concerns apply to care planning and communication. The Five Provisions and Welfare Aims framework does not aggregate, score, or grade welfare impacts, nor does it require validated affective inferences, as it does not generate welfare conclusions.

### 4.4. Implications for Practice, Science, and Legislation

#### 4.4.1. For Practice

For those responsible for animal care, the care/welfare distinction has a direct practical implication; providing appropriate resources or competent management practices is not the end of the task. Good care requires attention to whether the animal experiences the intended affective consequences of the provisions supplied (i.e., welfare-focused care). The updated Five Provisions framework provides a practical structure for welfare-focused care: the provisions specify what to provide, and the welfare aims specify what the animal should experience as a result. Whether the welfare aims are being achieved can be monitored by observing the animal: their behaviour, demeanour, and bodily condition provide ongoing evidence of their experience. This is consistent with what UK veterinary practice now calls contextualised care: care adapted to the circumstances of the individual animal, their owner, and the wider context, rather than a single gold standard applied to every case [27]. The Five Provisions and Welfare Aims give the animal side of that judgement a structure, specifying what to provide and what the animal should experience as a result. For a practitioner, the five provisions work as principles for providing a good life. The first four set out what to provide. Provision 5 keeps that provision consistent over time and across everyone who interacts with the animal, and the welfare aims keep the animal’s experience as the measure of success.

The revision of Provision 4 from “Appropriate Behaviour” to “Appropriate Choices” has specific practical implications. It directs attention away from whether an animal is displaying a range of behaviours, which may include stereotypic, reactive, or constrained behaviours, and toward whether the animal has genuine opportunities to exercise agency: to make meaningful choices about how to interact with their environment, other animals, and people. It asks a different question of the animal’s conditions and is more likely to identify situations in which an animal’s behaviour appears adequate, but opportunities for agency are restricted.

For organisations communicating their welfare-focused approach to animal care, the updated Five Provisions framework, shown in full in Figure 2, is the appropriate tool. It gives organisations a way to describe what they provide (the provisions) and what those provisions should achieve for the animal (the welfare aims), preserving the distinction between care and welfare by making both elements visible. Organisations currently using the Five Domains structure to communicate care provisions would be better served by the updated Five Provisions framework.

Integrated Care also provides a natural location for cross-provision content, i.e., topics that span all four provisions rather than sitting within any single one, such as animal health and welfare planning, biosecurity, or continuity of care in team-based situations. This applies whether the framework is used in legislation, regulatory codes, private standards such as certification and industry schemes, or educational materials that communicate appropriate animal care. Provision 5 directs attention to: (1) care planning sustained across the animal’s life and adapting to life stage (“over time”); (2) communication and documentation between caregivers that ensures continuity (“across all those who interact with the animal”); and (3) organisational culture and training that produces consistent provision regardless of which person is delivering care at any moment (“culture of care”) [28,29].

Many organisations have invested in the Five Domains Model as a recognisable signal of quality in relation to animal welfare, and there is no reason to abandon that connection when describing their work. The distinction between welfare assessment and care planning or communicating does not require a choice about whether to use the Model’s name. It requires precision about what the name is being used to claim. For organisations conducting welfare assessments, language that specifies the use is accurate and informative: “welfare assessment using the Five Domains Model,” “Five Domains welfare assessment,” or “welfare assessment based on the Five Domains Model.” Each of these should signal that the affective translation step has been completed and Domain 5 conclusions have been derived from evidence gathered across Domains 1 to 4.

For organisations using the domain structure to organise welfare-focused care standards or communicate care provisions, equally precise language is available: “standards organised around the Five Provisions and Welfare Aims, derived from the Five Domains Model,” “Five Domains-aligned care programme,” or “animal care programme aligned with the Five Domains Model.” These formulations retain the Model’s name and signal its influence on how care is structured (i.e., welfare-focused), without implying that welfare assessments have been conducted. In sectors where “management” is the standard term, equivalent formulations apply: “Five Domains-aligned management practices,” or “management programme aligned with the Five Domains Model.”

The practical value of this precision is mutual. Organisations communicate more clearly what they have actually done, and the people who rely on that communication better understand what has been produced. An organisation that develops care standards or conducts care audits against Five Domains-aligned criteria provides something of real value. Describing that accurately, rather than as a welfare assessment, does not diminish it.

#### 4.4.2. For Welfare Science and Assessment

The distinction between care and welfare determines what is being assessed. If welfare is equated with care, assessment involves checking whether provisions have been supplied: a resource audit. If welfare is understood as the animal’s affective experience, assessment requires the reasoning process set out in Section 4.1, ending in Domain 5 conclusions [3,30,31]. An assessment that lists conditions across Domains 1 to 4 without completing the affective translation step is an assessment of care inputs, not an assessment of welfare. This is a valid and useful exercise, but it should not be called a welfare assessment.

#### 4.4.3. For Legislation and Policy

The language of “needs” remains the basis of most animal welfare legislation globally. The term has been effective in establishing that animals require active care, but it frames welfare in terms of what people provide rather than what the animal experiences. Needs framing also leaves limited space for positive experiences and for agency [10,51]. When the statutory question is whether a need has been met, the focus is naturally directed to what has been provided. The default interpretation in regulatory contexts tends toward the minimum necessary to maintain homeostasis or prevent illness. The animal’s mental experience is not absent from statute, but it is typically located on the offence side (e.g., prohibitions on causing unreasonable or unnecessary pain or distress/suffering) [52,53] rather than the standard-of-care side, where “needs” framing predominates.

Animals have an interest in exercising agency, and their welfare is impacted by their ability to do so [3,8,10,17,24]. Needs language struggles to capture agency, because needs are defined by what must be provided. Agency cannot be provided directly; it can only be enabled by providing meaningful choices. The provisions and welfare aims framework can express this: the provision specifies the conditions to create, and the welfare aim names the positive mental experiences those conditions are intended to support, including those associated with exercising agency.

However, “needs” language has a strength that “provisions” language lacks. “Needs” carries moral force because saying that an animal has a need creates an obligation to meet it (A. Quain, personal communication, 2026). The concept of needs can also be extended to positive states, so an animal can be said to need social contact or opportunities to exercise agency. The two framings work together rather than competing: needs language establishes the obligation, and the provisions and welfare aims specify what that obligation requires in terms of both care inputs and affective outcomes.

Supplementing needs language with the provisions and welfare aims framework would place both the care input and the affective outcome within the standard of care, rather than splitting them between the standard of care and the offence provisions. This gives legislators and regulators a basis for setting both minimum care standards and aspirational welfare targets. The provisions and welfare aims make the care inputs and affective outcomes required by the process visible and distinct. In legislative terms, supplementing needs language with the provisions and welfare aims framework would reflect what the Five Domains Model makes explicit in scientific terms: that providing appropriate resources and conditions is a duty of care [52], and that the purpose of that duty is to promote positive mental experiences and minimise negative ones [54].

## 5. Conclusions

This paper proposes an update to the Five Provisions and Welfare Aims framework and clarifies the distinction between animal care and animal welfare. The updated framework shifts attention from provision as an endpoint to affective experience as the standard for evaluating the adequacy of care (i.e., welfare-focused care). The revision of Provision 4 to “Appropriate Choices” directs attention to whether animals have genuine opportunities to exercise agency, a dimension of welfare with direct positive affective consequences when supported. The revision of Provision 5 to “Integrated Care” addresses a related inconsistency: positive mental experiences are the outcome of care, not a category of care itself. Naming Provision 5 “Integrated Care” makes its integrative role explicit, parallels the role of Domain 5 in the Model, and ensures the distinction between care and welfare is applied consistently across all five provisions. Welfare Aim 5 names the affective outcome that integrated care is intended to support: a balance of mental experiences in which positives predominate, with the animal’s overall experience as the standard against which the integration is judged. Describing the reasoning process that distinguishes welfare assessment from welfare-focused care gives organisations a basis for identifying which application best serves their purpose and for accurately communicating their use of the Model. The provisions and welfare aims framework offers an alternative to inadequacies of needs language alone, as it retains practical guidance while directing attention to the mental experiences that care is intended to support. Both the Five Domains Model and the Five Provisions are grounded in the same affective state orientation. Applying either framework as intended requires users to uphold the underlying commitment that welfare-focused care is the means, and good welfare is the end. Specifically, the aim is for animals to have a good life under human care, with positive experiences predominating. Understood together, the Five Provisions are principles for providing a good life, and the welfare aims state what that good life means for the animal.

## Figures and Tables

**Table 1:** The updated Five Provisions and Welfare Aims framework. Each Provision names a category of care input. The Provide column specifies what the Provision means in practical terms. Each Provision is paired with a Welfare Aim that specifies the affective outcome the input is intended to support for welfare-focused care. Welfare Aim 5 is the integrating aim and corresponds to Domain 5 of the Five Domains Model.

	Provision	Provide	Welfare Aim
**1**	**Good Nutrition**	Ready access to fresh/clean water and a complete and balanced diet to support life stage.	Minimise thirst and hunger and enable eating to be a pleasurable experience.
**2**	**Good Living Space**	Sufficient space, shade/shelter or suitable housing, good air quality, appropriate lighting and temperature, and appropriate substrates and resting areas.	Minimise discomfort and promote physical, thermal, and sensory comforts.
**3**	**Good Health**	Preventative care or rapid diagnosis and treatment of disease and injury, and conditions that foster good muscle tone, posture and cardiorespiratory function.	Minimise pain, breathlessness, nausea, and other unpleasant experiences and promote the pleasures of fitness, strength, and well-coordinated physical activity.
**4**	**Appropriate Choices**	Appropriately varied and explorable spaces, opportunities for compatible social contact, and predictable human interactions the animal can influence or avoid.	Minimise fear, anxiety, and other unpleasant experiences and promote the pleasure of making meaningful choices and acting on them, including interest, engagement, and a sense of control.
**5**	**Integrated Care**	Consistent delivery over time and across all those who interact with the animal.	Support a balance of mental experiences where positives predominate.

**Table 2:** The substantive revisions to the Five Provisions and Welfare Aims framework. Provisions 1 and 3, and their Welfare Aims, retain their substance from Mellor (2016), but their Welfare Aims have been reworded for consistency with the structural revisions. When the revisions are structural, they change what the framework does rather than only being how it is worded. Minor wording adjustments to the Provide column and to other Welfare Aims, made for consistency with the structural revisions, are not listed individually.

Element	Original (Mellor, 2016)	Revised (This Paper)	Rationale for Revision
Provision 1 (specification)	Ready access to fresh water and a diet to maintain full health and vigour.	Ready access to fresh/clean water and a complete and balanced diet to support life stage.	This shifts from an outcome-leaning phrase (“full health and vigour” is a desired state) to a more conventional nutritional standard (“complete and balanced diet to support life stage”). The shift removes language that arguably belongs in the Welfare Aim or in Domain 3 (health) and replaces it with language consistent with current veterinary nutrition framing (life-stage-appropriate complete and balanced diets).
Provision 2 (structural revision)	Provision 2 named “Good Environment” with the specification to “provide shade/shelter or suitable housing, good air quality and comfortable resting areas” and the aligned aim to “minimise discomfort and exposure and promote thermal, physical and other comforts.”	Provision 2 is renamed “Good Living Space” with the specification “Sufficient space, shade/shelter or suitable housing, good air quality, appropriate lighting and temperature, and appropriate substrates and resting areas” and is paired with a Welfare Aim (“Minimise discomfort and promote physical, thermal, and sensory comforts”).	Resolves ambiguity introduced by the 2020 revision of the Five Domains Model, which renamed Domain 2 “Physical Environment” to distinguish it from “interactions with the environment” within Domain 4. “Living Space” signals the physical and structural conditions covered by Domain 2. “Exposure” (a condition rather than an experience) removed from the minimise clause; “and other comforts” replaced with “sensory comforts.”
Provision 3 (specification)	Prevent or rapidly diagnose and treat disease and injury, and foster good muscle tone, posture and cardiorespiratory function.	Preventative care or rapid diagnosis and treatment of disease and injury, and conditions that foster good muscle tone, posture and cardiorespiratory function.	“Preventative care” is a category of care provision, whereas “prevent” is something the animal might or might not be free of (a freedom, not a provision).
Provision 4 (header and specification)	Provision 4 named “Appropriate Behaviour” with the specification to “provide sufficient space, proper facilities, congenial company and appropriately varied conditions.”	Provision 4 is renamed “Appropriate Choices” with the specification “Appropriately varied and explorable spaces, opportunities for compatible social contact, and predictable human interactions the animal can influence or avoid.”	Reflects the 2020 revision of the Model, which formalised three categories of interaction within Domain 4, and the 2023 operationalisation of agency through choice, control, and challenge. “Choices” sits on the care side of the distinction; behaviour is an output of the animal, not an input the caregiver provides. The 2026 version explicitly names all three of Domain 4’s subcategories: environment (“varied and explorable spaces”), other animals (“compatible social contact”), and people (“human interactions the animal can influence or avoid”).
Welfare Aim 4	Minimise threats and unpleasant restrictions on behaviour and promote engagement in rewarding activities.	Minimise fear, anxiety, and other unpleasant experiences and promote the pleasure of making meaningful choices and acting on them, including interest, engagement, and a sense of control.	“Threats” describes a feature of the input rather than an affective outcome and is structurally inconsistent with the form of the other Welfare Aims. Replaced with the affective consequences (fear, anxiety) that restrictions on agency generate. The promote clause aligns with the operationalisation of agency through choice, control, and challenge and draws on the affective targets named in Mellor (2016).
Provision 5 (structural revision)	Provision 5 named “Positive Mental Experiences” with the specification to “provide safe, congenial and species-appropriate opportunities to have pleasurable experiences” and the aligned aim to “promote various forms of comfort, pleasure, interest, confidence and a sense of control.”	Provision 5 is renamed “Integrated Care” with the specification “Consistent delivery over time and across all those who interact with the animal” and is paired with a Welfare Aim (“Support a balance of mental experiences where positives predominate”).	The original Provision 5 named an affective outcome rather than a care input, breaking the structural pattern of Provisions 1 to 4. The revised Provision 5 names a care input (the integrated delivery of the first four provisions) and is paired with a Welfare Aim that names the affective outcome. Welfare Aim 5 plays the same integrative role within the Provisions framework as Domain 5 plays in the Model: it is the point where the affective outcome of the first four elements is brought together, rather than a fifth element in its own right.

## Data Availability

Not applicable.

## Abbreviations

The following abbreviations are used in this manuscript:

SPCA	Society for the Prevention of Cruelty to Animals
ZAA	Zoo and Aquarium Association Australasia
WAZA	World Association of Zoos and Aquariums
CPD	Continuing Professional Development
NZ	New Zealand
